# Mutations in the TMCO3 Gene are Associated with Cornea Guttata and Anterior Polar Cataract

**DOI:** 10.1038/srep31021

**Published:** 2016-08-03

**Authors:** Peng Chen, Xiaodan Hao, Wenfeng Li, Xiaowen Zhao, Yusen Huang

**Affiliations:** 1 State Key Laboratory Cultivation Base, Shandong Provincial Key Laboratory of Ophthalmology, Shandong Eye Institute, Shandong Academy of Medical Sciences, Qingdao, Shandong Province 266071, China; 2 Department of Oncology, the Affiliated Hospital of Qingdao University Medical College, Qingdao, China

## Abstract

The molecular basis for cornea guttata and anterior polar cataract remains idiopathic in most cases. In this study, our aim was to identify the disease-associated gene in Chinese patients with these conditions. Patients with the conditions from two Chinese families, and ten sporadic patients, were investigated. Genome-wide linkage and exome sequencing analyses showed transmembrane and coiled-coil domain 3 (TMCO3) as the disease candidate gene for a coding heterozygous mutation c.41C > T, resulting in a P14L amino acid change that co-segregated with the disease phenotype as discovered in Family A. TMCO3 belongs to the monovalent cation: protein antiporter 2 transporter family, a moderately large group whose members all share a very similar function under normal physiological conditions. The gene is expressed in the human cornea, lens capsule, and choroid-retinal pigment epithelium. This study reveals, for the first time, that mutations in TMCO3 are associated with cornea guttata and anterior polar cataract, warranting further investigation into the pathogenesis of this disorder.

Fuchs’ corneal dystrophy (FCD; MIM 136800), which was first described by the Austrian ophthalmologist Ernst Fuchs in 1910, is a common hereditary disease of the corneal endothelium^1^,^2^,^3^. The first clinical signs appear in approximately the fourth decade of life with the formation of excrescences in the central Descemet membrane, termed guttae (Latin: drops), eventually progressing, in some cases, to corneal edema^4^,^5^. The prevalence of FCD is generally considered to be around 4% in individuals aged over 40, although higher estimates have been reported in some populations, such as 9%, 6.5%, and 21.6% in Icelandic, Singaporean, and inbred American populations, respectively^6^,^7^,^8^,^9^.

FCD is typically inherited in an autosomal dominant fashion, although females are more frequently affected than males by a two-to-one ratio, precluding strictly Mendelian population-wide autosomal dominant inheritance with a high penetrance^1^,^2^,^10^,^11^,^12^,^13^. The pathological mechanism that results in FCD has not been established. Studies have shown that the disease is associated with mutations in SLC4A11 (OMIM 610206)^14^,^15^, LOXHD1 (OMIM 613072)^16^, TCF8/ZEB1 (OMIM 189909)^17^,^18^ and COL8A2 (OMIM 120252)^19^,^20^,^21^ and loci on chromosomes 13,18,5, and 9, where the genes have not yet been identified. Baratz and colleagues described the results of a genome-wide association study (GWAS) that revealed a highly significant association between single nucleotide polymorphisms (SNPs), most notably rs613872, in the transcription factor 4 (TCF4) gene and typical, late onset FCD^22^. The TCF4 gene at 18q21 (also known as ITF2 and SEF-2) codes for the helix-loop-helix transcription factor E2-2. A large, non-coding trinucleotide repeat expansion in introns of the TCF4 gene is responsible for most cases of FCD^23^,^24^. FCD may occur independently or in association with other ocular or systemic abnormalities.

In our previous study, cornea guttata were found in 17 out of 2,026 patients with cataract^25^. Cornea guttata with anterior polar cataract (OMIM 121390) is a rare autosomal dominant inherited disease that was initially described by Ichikawa and Hiraga in 1951^26^. It remains to be clarified whether the phenotype of cornea guttata with anterior polar cataract is related to specific cataract gene alleles or closely linked modifiers. In this study, we investigated two families affected by this condition and ten sporadic cases and performed genome-wide linkage and exome sequencing analyses in one family.

## Results

### Clinical assessment and findings

**Figure 1:**
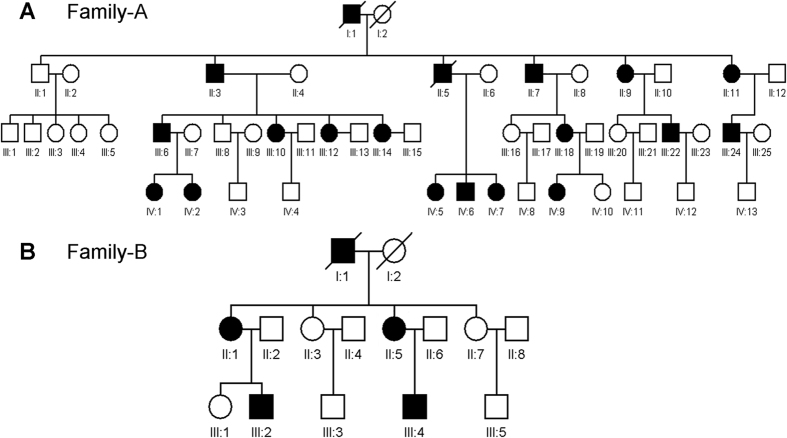
Pedigrees of the two Chinese families with dominant cornea guttata and anterior polar cataract. Affected men and women are indicated by filled squares and circles, respectively. Normal individuals are shown as empty symbols. Deceased individuals are indicated with slashes (/).

**Figure 2:**
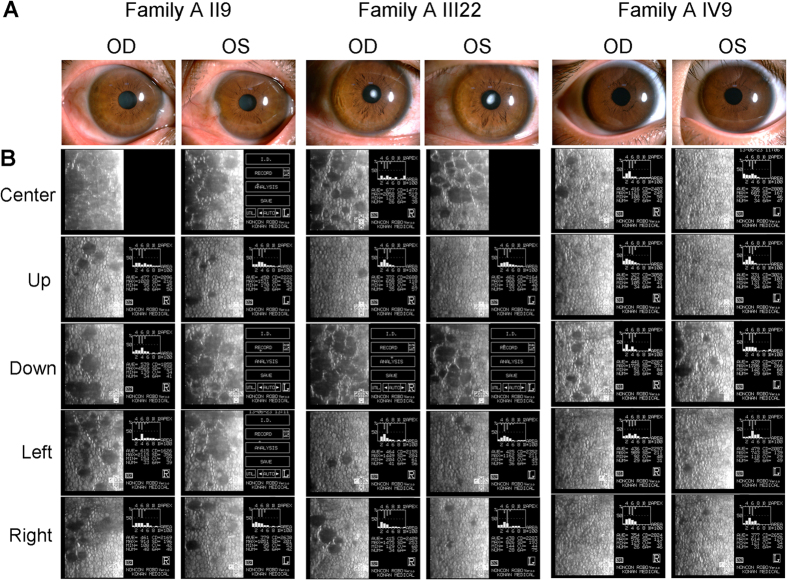
Slip-lamp and specular microscopy photographs of the affected individuals in Family (**A**). OD: right eye, OS: left eye.

In this section, we will describe two Han Chinese families from Qingdao that are affected by monogenic cornea guttata and anterior polar cataract with a dominant inheritance model (Fig. 1). Under ophthalmic examination, 17 out of 52 members of Family A, a four-generation family, were identified as being affected by cornea guttata and anterior polar cataract (Figs 1A and 2). Another two patients in this family were deceased.

**Figure 3:**
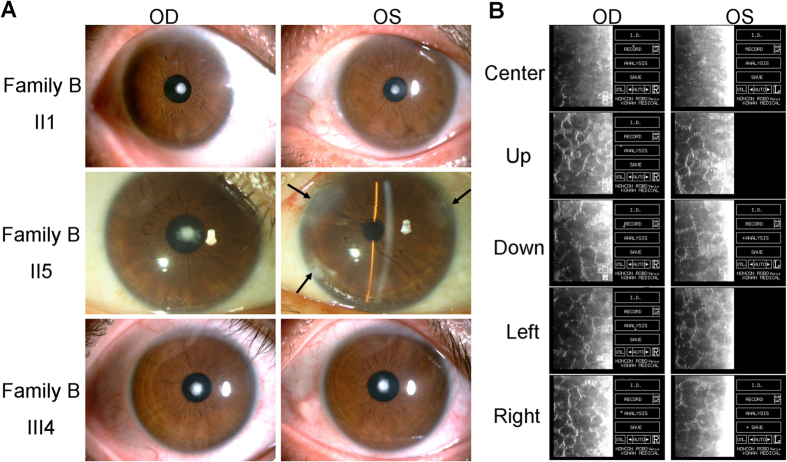
Slip-lamp and specular microscopy photographs of the affected individuals in Family (**B**). OD: right eye, OS: left eye. The arrowhead indicates the position of the operative incision in the phacoemulsification and intraocular lens implantation surgery.

The clinical features of patients in Family A were presented in Supplementary Table 1. Four affected individuals (Figs 1B and 3) were found among the 13 examined family members in the three-generation Family B, which also had one deceased patient.

The two families in our study had some common features, such as FCD. There was no family history of other systemic abnormalities. Most corneal changes were bilateral, equally advanced in both eyes, and limited to the posterior cornea. The degree of severity varied between patients, although in all subjects, the central cornea was more significantly affected than the periphery (Figs 2B and 3B). Polar cataract was not always evident at birth, but often deteriorated after middle age. II7 and III6 in Family A received phacoemulsification and intraocular lens implantation surgery at the ages of 56 and 37, respectively. II5 in Family B, meanwhile, underwent these procedures at the age of 46. Cornea guttata progressed slowly with age, but it seemed that no corneal edema was found in all patients.

### Genome-wide linkage analysis to identify causal regions

We performed genome-wide linkage and exome sequencing analyses in the multi-generational Family A. First, we genotyped eight individuals (II3, II7, II9, III8, III18, III22, IV9, and IV12) using Illumina Human OmniZhonghua8 BeadChip. Multipoint parametric linkage analysis was performed in Merlin by using pruned autosomal SNPs and assuming a dominant inheritance of the disease phenotype. LOD scores were obtained by multipoint linkage analysis at a recombination rate of 0.0001 under an autosomal dominant model with 100% penetrance. Seven loci were positive, but non-significant LOD scores (>3) were identified (Table 1). LOD scores (0-1, <0, < −2) obtained by multipoint linkage analysis were present in Supplementary Table 2. The maximum possible LOD score, 2.0, could be achieved by genotyping eight individuals (six affected and two normal). We focused on these candidate regions in Table 1 in order to search for causative variants using exome sequencing.

We performed the genotyping data of a few other SNPs of other genes in the 2.9 Mb chromosome 13q34 interval. These SNPs includes rs117848947, rs36001240, rs9577843, rs383353, rs282613, rs946006, rs9549573, rs12855893, rs3024770, rs3024771, rs2297186, rs3794423, rs2270393, rs2302758, rs185071949, rs117209253 and rs2274714. We identified rs2302758-rs2274714 (1.924M, chr13: 113326149-114021545) with a maximum LOD score of 5.282 (Supplementary Table 3).

### Exome sequencing analysis to explore TMCO3 as the associated gene

We then performed exome sequencing analysis on five affected individuals (II3, II7, III18, III22, and IV9) from Family A (Fig. 1A). An average of 6.13 billion bases of sequences was generated for each individual. BWA was invoked to map the reads to the hg19 human reference genome^27^. On average, each sample had a mean depth of 56.1 and 86.2% of the exome sequences were covered at 10X or more. GATK Unified Genotyper, with the recommended filtering criteria, was invoked to call single nucleotide variants (SNV) and indels^28^.

After variant annotation, we focused our analysis primarily on nonsynonymous variants (missense, nonsense, and read-through), splice acceptor and donor site variants, and coding Indels, on the basis that synonymous variants were less likely to be pathogenic. Using the public databases, dbSNP, 1000 Genomes Project, HapMap 8 database, and YH database, we filtered our exome data.

Given that familial cornea guttata and anterior polar cataract with an autosomal dominant inheritance pattern is rare, we assumed that the variants were also rare. Taken together, we focused on novel and less common variants (minor allele frequency of less than 0.05 in the 1000 Genomes Project), filtering the data accordingly (Table 2).

**Figure 4:**
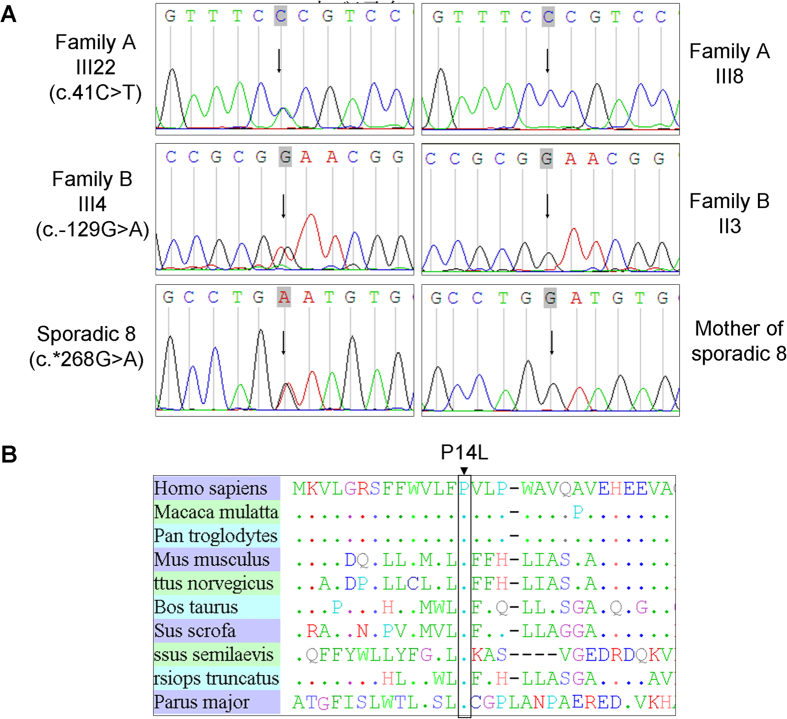
Sequence chromatograms of all TMCO3 mutations identified in the study. (**A**) The left chromatogram represents the sequences of the affected individuals, the right chromatogram represents the sequence of a normal family member, and the arrow indicates the location of the mutations. (**B**) Alignment of sequences surrounding the P14L mutation in Homo sapiens, Macaca mulatta, Pan troglodytes, Mus musculus, Rattus norvegicus, Bos taurus, and Sus scrofa. Proline on the 14th position in TMCO3 is highly conserved among different species, including Homo sapiens (NP_060375.4), Macaca mulatta (AFI33644.1), Pan troglodytes (XP_001145898.1), Mus musculus (NP_758486.1), Rattus norvegicus (XP_225016.4), Bos taurus (XP_586612.1), Sus scrofa (NP_001254780.1), Cynoglossus semilaevis (XP_008308536.1), Tursiops truncates (XP_004319523.1), and Parus major (XP_015476001.1).

Segregation was then analyzed by applying Sanger sequencing to the four non-synonymous SNPs, one UTR, and 51 indels with the 49 members of Family A. Only one single nucleotide variant, chr13:113495622, that was heterozygous in the 17 affected individuals, lay within the critical linkage regions defined by haplotype co-segregation analysis. This coding mutation (c.41C > T; p. P14L) was in the exon 1 of TMCO3 (Table 3, Fig. 4A). The mutation was not found in the 200 exomes of Chinese origin.

The c.41C > T generated a missense mutation (p. P14L). Multiple alignments of Pro14 of the human TMCO3 protein from different species revealed 100% identity, suggesting that it was highly conserved during evolution (Fig. 4B). The SIFT tool analysis predicted that the replaced amino acid was “damaged” to protein function.

### Sanger sequencing to verify the candidate gene TMCO3

To confirm TMCO3 as the gene associated with cornea guttata and anterior polar cataract, we used Sanger sequencing to screen all members of Family B. All four clinically affected subjects, but none of those who were unaffected in Family B, carried the heterozygous mutation c.−129G > A in the 5′ UTR of TMCO3 (Table 3, Fig. 4A). The heterozygous mutation c.−129G > A in TMCO3 completely co-segregated with the dominant cornea guttata and anterior polar cataract phenotype within Family B.

We then carried out direct Sanger sequencing of the TMCO3 exons in the 10 unrelated (based on their self-identified geographical ancestry), sporadic patients with cornea guttata and anterior polar cataract. A heterozygous mutation (c.^*^268 G > A) in the 3′ UTR of TMCO3 was identified in one patient (Table 3, Fig. 4A). In the parents of the sporadic patient (sporadic 8), no variant in the TMCO3 gene was detected upon sequencing the TMCO3 exons.

### TMCO3 expression analysis

To get an insight into the expression of TMCO3 in human eye tissues, we performed an extensive examination of human expressed sequences located in the NCBI UniGene database (http://www.ncbi.nlm.nih.gov/unigene/) and the Eyebrowse site (http://eyebrowse.cit.nih.gov/), which displayed expressed sequence tags (ESTs) obtained from complementary DNA clones of eye tissues derived from NEIBank and other sources. We identified 12 ESTs out of about 235 matching the TMCO3 gene in different parts of the eye, such as the human primary ocular pericytes, cornea, retina, retina foveal and macula, choroid, and retinal pigment epithelium (RPE).

**Figure 5:**
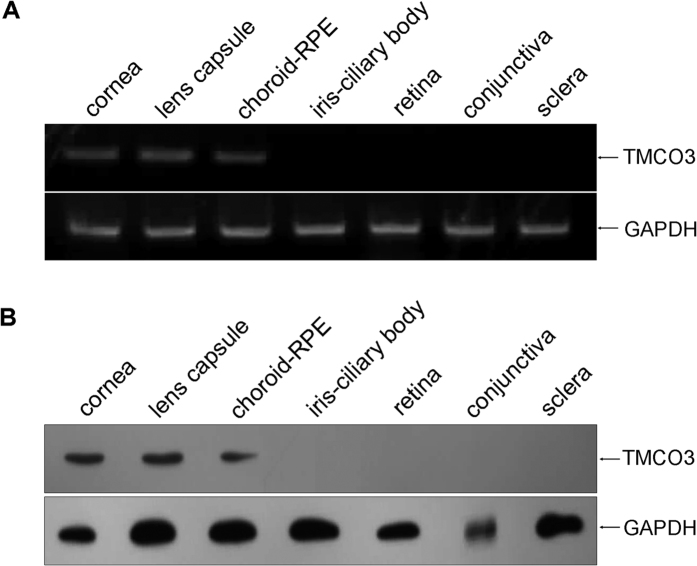
TMCO3 expression in human ocular tissues. (**A**) mRNA expression of TMCO3 in the human cornea, conjunctiva, ICB, sclera, retina, choroid-REP, and lens capsule. (**B**) TMCO3 expression in the human cornea, conjunctiva, ICB, retina, choroid-REP, and lens capsule by Western blotting. ICB: iris-ciliary body; RPE: retinal pigment epithelium.

**Figure 6:**
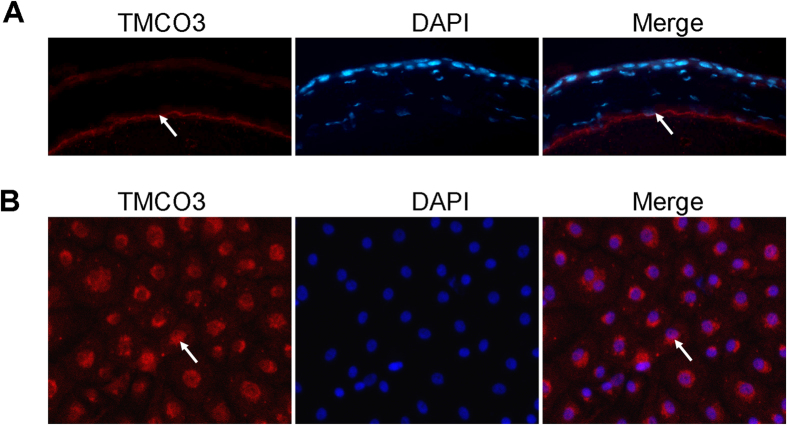
Representative figures of immunofluorescence staining of TMCO3 in mouse and rabbit corneal endothelial cells. The arrowhead indicates the expression of TMCO3. Magnification: 400×.

To ensure that TMCO3 was expressed in the eye, we examined TMCO3 expression in different human ocular tissues using RT-PCR and Western blotting. The TMCO3 gene and protein were expressed in the human cornea, lens capsule, and choroid-RPE (Fig. 5). In rabbit corneal endothelial cells, TMCO3 was detected in the cytoplasm by immunofluorescence microscopy (Fig. 6).

## Discussion

Cornea guttata with anterior polar cataract (121390) is rarely seen. Dohlman^26^ described a Swedish family in which 15 members had corneal endothelial dystrophy (cornea guttata) in association with anterior polar cataract. In all patients, the central cornea was more severely affected than the periphery. Three other members had either endothelial dystrophy or polar cataract. It also appeared that a number of deceased family members probably had, at least, polar cataract. The onset ages were three and ten years, and the disease became stationary after puberty and progressed slowly, resulting in a ‘beaten metal’ appearance on retro-illumination.

Dohlman^26^ commented that the changes were limited so sharply to the posterior of the cornea that barely more than the endothelium and Descemet membrane could feasibly be involved. The corneal changes were roughly equally advanced in both eyes of most patients; however, the severity of changes varied. The lesions were most advanced in the central cornea and decreased in severity toward the periphery. In young patients, impairment of vision was related to polar cataract, but with advancing age, corneal deterioration contributed more to the visual disability.

Traboulsi and Weinberg^29^ observed an American family in which 12 members had both abnormalities under discussion. All affected individuals had excellent visual acuity. They were descended from a family that had emigrated to the United States in the 17th century from Ireland, where they had settled in the 13th century after moving from Scandinavia.

In our study, the symptoms of the patients in Family A and Family B were not the same as described previously. Clinically, polar cataract did not seem to be evident at birth, but tended to deteriorate after middle age in these patients. Three of them underwent phacoemulsification and intraocular lens implantation at middle age. Cornea guttata progressed slowly, but there seemed to be no corneal edema in any of the patients.

In the past several decades, identification of genes that underlie disease in a monogenic form has primarily been through the selection of candidate genes for testing or by the use of positional cloning. The former approach requires prior knowledge of the pathogenesis of a disease for gene selection. Linkage analysis is an effective method of detecting susceptibility loci with a large effect size^30^. A combination of linkage analysis and Sanger sequencing has been used to search for causative variants of diseases^31^. However, due to locus and disease heterogeneity, the conventional approach has proven difficult to perform, as the causative variants may be present in any number of candidate genes. The recent development of massively parallel DNA-sequencing technologies provides a powerful means of identifying causative variants that are responsible for Mendelian or common disorders^32^. Although exomes only constitute about 1% of the human genome, they are estimated to be the major source of causative variants, constituting 85% of disease-causing mutations^33^. Therefore, by combining traditional linkage analysis and whole-exome sequencing, we could maximize our chances of identifying a causative variant.

In this study, we identified a novel dominant gene associated with cornea guttata and anterior polar cataract, TMCO3, which had different heterozygous mutations in two families with autosomal dominant cornea guttata and anterior polar cataract (c.41C > T, c.−129G > A). Another heterozygous mutation (c.−129G > A) was identified from the sporadic patients. Parental clinical information of the ten sporadic patients was gathered, and no ocular abnormality or family history of other systemic abnormalities was found. In the parents of the sporadic patient 8, no variants in the TMCO3 gene were detected upon sequencing the TMCO3 exons.

TMCO3, also known as putative LAG1-interacting protein, is a 677 amino acid multi-pass membrane protein that probably functions as a Na+/H+ antiporter. TMCO3 belongs to the monovalent cation: protein antiporter 2 transporter family, a moderately large family whose members all share a very similar function under normal physiological conditions.

Ion transporters play an important role in the physiological function of corneal endothelial cells. The major ion transporters that significantly regulate fluid transport are sodium bicarbonate cotransporter-1; Na+: 2HCO_3_− (pNBCe1) and sodium proton exchanger-1; and Na+/H+ (NHE1). Also postulated as affecting fluid secretion are the monocarboxylate transporters (MCTs): lactate: H+) 1, 2, and 4 34,35 and SLC4A11, a recently characterized intracellular pH regulator^36^,^37^. Decreased membrane expression of Na+/K+ ATPase^38^,^39^ and downregulation of SLC4A11^40^ in FED have been reported previously. Loss of ion transport, due to the decreased expression or loss of cell density, leads to fluid retention, stromal edema, and loss of corneal transparency. The functionality of corneal endothelial cells, which maintain stromal hydration and corneal transparency, is challenged by FED.

It is also understood that the bulk of the lens comprises tightly packed fiber cells layered one on top of the other, but there are questions regarding which cells form the cellular barrier that enables the lens to maintain internal sodium and potassium concentrations that are distinct from the concentrations found in aqueous or vitreous humor. The monolayer of epithelium that covers the anterior lens surface has a high Na,K-ATPase activity compared to lens fibers. The epithelium appears to be specialized for active sodium-potassium transport and probably contributes to the maintenance of ion balance in the entire fiber mass. In the epithelium monolayer, Na,K-ATPase activity is highest at the periphery and lowest at the center of the anterior pole of the lens^41^,^42^.

In the current study, the TMCO3 protein was expressed in human cornea, lens capsule, choroid-retinal pigment epithelium, mouse corneal endothelial cells, and rabbit corneal endothelial cells. It may be involved in the physiological function of corneal endothelial cells and lens epithelial cells. However, the exact mechanism of TMCO3 action and its role in the pathogenesis of dominant cornea guttata and anterior polar cataract remain unclear, and future functional studies will be important in exploring this. To date, there have been no documented studies on the TMCO3 gene in eye development and the data in our study indicating its involvement in a devastating eye disease provides excellent motivation for future investigation, which, in turn, should enable a dissection of the gene’s relationship with the pathogenesis of dominant cornea guttata and anterior polar cataract.

## Methods

### Ethics statement

This study was performed in accordance with the Declaration of Helsinki and approved by the Ethics Committee of Shandong Eye Institute (Qingdao, China). Written informed consent was obtained from all participants (or their guardians).

### Study population

Two Han Chinese families (designated as Family A and Family B) with dominant cornea guttata and anterior polar cataract, ten sporadic patients with cornea guttata and anterior polar cataract and their parents, and 200 matched, normal controls were included. The ten sporadic patients and their parents, and the 200 controls of Han Chinese ethnicity, were non-related Qingdao locals recruited at the Qingdao Eye Hospital, Shandong Eye Institute (Qingdao, China). All participants underwent an extensive, standardized examination by ophthalmologists. The diagnosis was confirmed by ophthalmic examinations, including visual acuity, slit-lamp microscopy, tonometry, keratometry, specular microscopy, ultrasonic A/B scan, and a history of cataract extraction, if any. Ocular photographs were taken using slit-lamp photography. There was no family history of other systemic abnormalities in Family A, Family B, or the ten sporadic patients.

### Genome-wide linkage analysis

For the genome-wide linkage analysis, approximately 50 ng of genomic DNA of the eight individuals (II3, II7, II9, III8, III18, III22, IV9, and IV12) from Family A was used for the genotyping analysis by Illumina HumanOmniZhongHua-8 BeadChip. Briefly, each sample was whole-genome amplified, fragmented, precipitated, and re-suspended in an appropriate hybridization buffer. Denatured samples were hybridized on prepared Illumina HumanOmniZhongHua-8 BeadChip. After hybridization, the BeadChip oligonucleotides were extended by a single labeled base, which was detected by fluorescence imaging with an Illumina Bead Array Reader. Normalized bead intensity data obtained for each sample was loaded into the Illumina GenomStudio software, which converted fluorescence intensities into SNP genotypes. After quality control filtering, 872,261 SNPs were retained for linkage analysis. A familial relationship check, based on IBD sharing, was carried out to confirm the collected pedigree information. Multipoint parametric linkage analysis was performed in Merlin by using the pruned autosomal SNPs and assuming dominant inheritance with a disease allele frequency of 0.0001 and penetrance rate of 1.

### Whole exome capture and library construction

Exome sequencing was performed on five patients (II3, II7, III18, III22, and IV9) at Berry Genomics Co. Ltd., Beijing, China. Venous blood (5 ml) was collected from the participants and the total human genomic DNA was isolated with the DNA isolation kit for mammalian blood (Tiangen, Beijing, China). Venous blood and genomic DNA samples were stored at −80 °C before use. The Human All Exon (50 Mb) target enrichment system (Agilent Technologies, Santa Clara, CA, USA) was used for whole exome capture and the HiSeq 2500 Sequencing System (Illumina Inc., San Diego, CA, USA) was used for massive parallel sequencing. The gene sequences for this array are available from the Consensus Coding Sequence Region (CCDS) database (http://www.ncbi.nlm.nih.gov/projects/CCDS/).

### Variant analysis

The sequencing reads were aligned to the human reference genome (NCBI Build 36.3). Alignment of the sequences from three affected individuals was performed using the SOAP aligner after the duplicated reads were removed^43^ and SNPs were called using the SOAP snp set with default parameters^44^. Indels affecting the coding sequence or splicing sites were identified, as described previously^45^.

Data was provided as lists of sequence variants (SNPs and short indels), relative to the reference genome. Identified variants were filtered against the Single Nucleotide Polymorphism database (dbSNP, http://www.ncbi.nlm.nih.gov/projects/SNP/snp_summary.cgi/), 1000 Genomes Project (http://www.1000genome.org/), HapMap 8 (http://hapmap.ncbi.nlm.nih.gov/) database, and YH database^46^.

### Verification of variants

Sanger sequencing was used to determine whether any of the remaining variants co-segregated with the disease phenotype in Family A. Primers flanking the candidate loci were designed, based on genomic sequences of Human Genome (hg18/build36.3), and synthesized. After filtering, all shared variants of the three affected individuals were then confirmed by PCR and analyzed on an ABI 3730XL Genetic Analyzer. Sequencing data were compared in a pair-wise manner with the Human Genome database.

Afterwards, we sequenced all the exons and flanking introns of the TMCO3 gene (NM_017905.4) in the patients from Family B and the ten sporadic patients to detect other mutation sites, using the Sanger sequencing method. As an additional step, the detected variants were sequenced in the 200 normal control subjects.

### Reverse-transcription polymerase chain reaction (RT-PCR)

Total RNA was prepared from venous blood (0.2 ml) of the family members, using the RNA isolation kit for mammalian blood (Tiangen, Beijing, China). Total RNA was prepared from each human ocular tissue, using the NucleospinRNA kit (BD Biosciences, Palo Alto, CA, USA), and reverse-transcribed into first-strand cDNA, using the Primescript™ First-Strand cDNA Synthesis kit (TaKaRa, Dalian, China). Gene-specific cDNA fragments were amplified with DNA polymerase (Tiangen, Beijing, China). The expression of genes was normalized to glyceraldehyde-3-phosphate dehydrogenase (GAPDH). The primer sequences used for TMCO3 were forward-5′-AGGTGATAAAGAAGCAGGTGACATC-3′ and reverse-5′-GCAGCTAGCGAAAAGAGAATTTG-3′, while for GAPDH they were forward-5′- ACCACAGTCCATGCCATCAC-3′ and reverse-5′-TCCACCACCCTGTTGCTGTA-3′. PCR amplification products were analyzed using agarose gel electrophoresis.

### Immunofluorescence staining

Eyeballs were snap-frozen in a Tissue-Tek optimum cutting temperature compound (Sakura Finetechnical, Tokyo, Japan). For immunofluorescence staining, frozen sections or cultured cells were fixed by 4% paraformaldehyde, permeabilized with Triton X-100, and blocked with normal serum. The samples were stained with primary antibodies overnight and subsequently with fluorescein-conjugated secondary antibodies for one hour. All stainings were examined under an Eclipse TE2000-U microscope (Nikon, Tokyo, Japan) after being counterstained with 4′,6-diamidino-2-phenylindole.

Rabbit corneal endothelial cells were plated in a glass culture dish (Biousing Biotech Co., Wuxi, China) at a density of approximately 4.0 × 10^4^ cells/35-mm dish in a complete medium, and grown to subconfluence. After the medium was removed, the cells were fixed in 4% paraformaldehyde in PBS for 15 minutes, followed by three PBS washes. The fixed cells were incubated with 0.2% Triton X-100 in PBS for five minutes, blocked in 5% BSA, and incubated with an anti-TMCO3 antibody (Abcam, Cambridge, MA, USA) for 30 minutes, before incubation with the fluorescence-conjugated secondary antibody for one hour. Images were obtained using an Eclipse TE2000-U confocal laser scanning microscope (Nikon, Tokyo, Japan).

## Additional Information

**How to cite this article**: Chen, P. *et al*. Mutations in the TMCO3 Gene are Associated with Cornea Guttata and Anterior Polar Cataract. *Sci. Rep*. **6**, 31021; doi: 10.1038/srep31021 (2016).

## Supplementary Material

Supplementary Information

## Figures and Tables

**Table 1:** LOD scores (>1) obtained by multipoint linkage analysis at a recombination rate of 0.0001 under an autosomal dominant model with 100% penetrance.

chr	start SNP	start SNP position	end SNP	end SNP position	maximum LOD
1	rs11121675	11060075	rs10803420	13954853	1.793
5	kgp869472	75590805	rs1922495	103478518	1.8
11	rs12420917	122477025	rs11221249	128196675	1.8
13	kgp2046536	112195456	rs9562200	115086044	1.798
17	kgp8173775	36975	rs11650689	1244992	1.77
18	kgp5379418	2335224	rs7237881	2634943	1.09
19	kgp2761736	56583279	rs917649	56924761	1.2

**Table 2:** Genetic variants identified in the five patients (II3, II7, III18, III22, and IV9) of Family A through exome resequencing.

Filter (not in 1000 Genomes Project (MAF ≥ 0.05), the dbSNP, HapMap 8, or YH database)	Genetic variants					
	SNV: Exon	SNV: Splicing site	SNV: Intron	SNV: UTR	Indel	Total
II3	721	18	577	115	2257	3688
II7	831	26	619	117	2092	3685
III18	832	17	550	130	1845	3374
III22	697	21	545	94	1887	3244
IV9	832	21	725	127	2156	3861
Heterozygous mutations shared by five affected individuals Non-synonymous In the linkage regions	4	0	0	1	51	56

**Table 3:** Genetic variants identified in TMCO3 in the two Chinese families and a sporadic patient with cornea guttata and anterior polar cataract.

Patient	Chromosome/Position/Gene name	dbSNP rs# cluster id	Mutation type	Codons	Substitution
Family A	chr13/113495622/TMCO3	rs185071949	Missense	c.41C > T	P14L
Family B	chr13/113491225/TMCO3	Novel	5′ UTR	c.-129G > A	
Sporadic 8	chr13/113549806/TMCO3	rs184333178	3′ UTR	c.*268 G > A	
