# Supplementary material for: Mutations in the TMCO3 Gene are Associated with Cornea Guttata and Anterior Polar Cataract
Source: Sci Rep. 2016 Aug 3;6:31021. doi: 10.1038/srep31021 (PMC4971526; doi:10.1038/srep31021)
Supplement: Supplementary Information [file srep31021-s1.doc]

**Mutations in the TMCO3 Gene are Associated with Cornea Guttata and Anterior Polar Cataract**

Peng Chen^1^, Xiaodan Hao^1^, Wenfeng Li^2^, Xiaowen Zhao^1^, Yusen Huang^1^*

1 State Key Laboratory Cultivation Base, Shandong Provincial Key Laboratory of Ophthalmology, Shandong Eye Institute, Shandong Academy of Medical Sciences, Qingdao, Shandong Province 266071, China.

2 Department of Oncology, the Affiliated Hospital of Qingdao University Medical College, Qingdao, China

PC and XH contributed equally to the work presented here and should therefore be regarded as equivalent authors.

*Correspondence: huang_yusen@126.com

**Supplementary Table** **1.** The clinical features of patients with cornea guttata and anterior polar cataract in Family A.

| Patient | Age (years) / Gender | Eye | Visual  acuity | Best  corrected  visual acuity | Lens | Iop (mmHg) | Surgery and  trauma history |
| --- | --- | --- | --- | --- | --- | --- | --- |
| II3 | 78/M | OD | 0.05 | 0.05 | Cataract | 16 | No |
|  |  | OS | 0.05 | 0.1 | Cataract | 15 | No |
| II7 | 67/M | OD | 0.5 | 0.6 | IOL | 14 | Phacoemusification+IOL |
|  |  | OS | 0.4 | 0.5 | IOL | 12 | Phacoemusification+IOL |
| II9 | 63/F | OD | 0.6 | 0.6 | Anterior polar cataract | 18 | No |
|  |  | OS | 0.5 | 0.8 | Anterior polar cataract | 16 | No |
| II11 | 60/F | OD | 0.5 | 0.5 | Cataract | 18 | No |
|  |  | OS | 0.2 | 0.5 | Cataract | 16 | No |
| III6 | 48/M | OD | 0.2 | 0.6 | IOL | 14 | Phacoemusification+IOL |
|  |  | OS | 0.2 | 0.6 | IOL | 14 | Phacoemusification+IOL |
| III10 | 47/F | OD | 0.2 | 0.5 | Normal | 15 | No |
|  |  | OS | 0.5 | 0.5 | Normal | 17 | No |
| III12 | 46/F | OD | 0.2 | 0.6 | Normal | 14 | No |
|  |  | OS | 0.5 | 0.6 | Normal | 15 | No |
| III14 | 35/F | OD | 0.3 | 0.5 | Normal | 16 | No |
|  |  | OS | 0.5 | 0.5 | Normal | 13 | No |
| III18 | 34/F | OD | 0.5 | 0.8 | Normal | 17 | No |
|  |  | OS | 0.8 | 1.0 | Normal | 18 | No |
| III22 | 39/M | OD | 0.3 | 0.5 | Anterior polar cataract | 13 | No |
|  |  | OS | 0.6 | 0.6 | Anterior polar cataract | 14 | No |
| III24 | 34/M | OD | 0.6 | 0.8 | Normal | 16 | No |
|  |  | OS | 0.5 | 0.8 | Normal | 16 | No |
| IV1 | 23/F | OD | 0.4 | 0.8 | Normal | 12 | No |
|  |  | OS | 0.5 | 0.9 | Normal | 13 | No |
| IV2 | 21/F | OD | 0.3 | 0.9 | Normal | 15 | No |
|  |  | OS | 0.6 | 0.9 | Normal | 14 | No |
| IV5 | 44/F | OD | 0.5 | 0.9 | Normal | 11 | No |
|  |  | OS | 0.6 | 0.8 | Normal | 13 | No |
| IV6 | 41/M | OD | 0.6 | 1.0 | Normal | 18 | No |
|  |  | OS | 0.5 | 1.0 | Normal | 19 | No |
| IV7 | 38/F | OD | 0.6 | 1.0 | Normal | 20 | No |
|  |  | OS | 0.6 | 1.0 | Normal | 19 | No |
| IV9 | 10/F | OD | 1.0 | 1.0 | Normal | 13 | No |
|  |  | OS | 1.0 | 1.0 | Normal | 13 | No |

**Supplementary Table** **2.** LOD scores (0-1, < 0, < -2) obtained by multipoint linkage analysis at a recombination rate of 0.0001 under an autosomal dominant model with 100% penetrance.

| chr | start SNP | end SNP | maximum LOD |
| --- | --- | --- | --- |
| 1 | kgp11435978 | kgp11071481 | < -2 |
| 1 | rs4908527 | kgp6061546 | < 0 |
| 1 | rs2245197 | kgp9169181 | 0.792 |
| 1 | kgp8870171 | kgp2417507 | < -2 |
| 2 | rs6712483 | rs6737791 | < -2 |
| 3 | rs6805411 | rs12636891 | < -2 |
| 4 | rs12511220 | kgp2961701 | < -2 |
| 5 | rs10076494 | rs13157620 | < -2 |
| 5 | rs4235665 | rs4235691 | < 0 |
| 5 | kgp6215565 | rs2770967 | < -2 |
| 6 | rs6916246 | rs2142521 | < -2 |
| 7 | rs7806249 | kgp1982129 | < -2 |
| 8 | rs2003497 | rs28859044 | < -2 |
| 9 | kgp2867527 | rs10780200 | < -2 |
| 10 | rs10904561 | rs9418986 | < -2 |
| 11 | rs10081 | rs585342 | < -2 |
| 11 | rs512932 | kgp6152779 | 0.766 |
| 11 | rs10893877 | rs10894947 | < 0 |
| 12 | kgp7445596 | rs11615340 | < -2 |
| 13 | rs1889574 | rs3783083 | <-2 |
| 13 | kgp12429885 | rs12020039 | 0.976 |
| 14 | kgp7281650 | rs17112078 | <-2 |
| 15 | kgp5725677 | rs7162261 | <-2 |
| 16 | rs2858016 | kgp5637931 | <-2 |
| 17 | kgp1471707 | rs7218436 | 0.605 |
| 17 | rs8080237 | kgp796957 | <-2 |
| 18 | kgp2812045 | rs1439425 | <-2 |
| 18 | rs312987 | kgp3653066 | 0.9 |
| 18 | kgp1185334 | rs8090956 | 0.65 |
| 18 | rs949306 | rs1519619 | <-2 |
| 19 | rs3745924 | rs11084381 | <-2 |
| 19 | kgp4010574 | kgp9294654 | 0.861 |
| 19 | rs145011 | kgp6135614 | 0.719 |
| 19 | kgp6860394 | kgp6860394 | <-2 |
| 19 | rs10413037 | rs7253514 | <0 |
| 20 | rs8122066 | rs6062357 | <-2 |
| 21 | kgp1775301 | rs2839368 | <-2 |
| 22 | rs361973 | rs715586 | <-2 |

Supplementary Table 3. LOD scores obtained by multipoint linkage analysis under an autosomal dominant model with 100% penetrance.

| POSITION | LOD | ALPHA | HLOD |
| --- | --- | --- | --- |
| rs117848947 | 1.926 | 1.000 | 1.926 |
| rs36001240 | 1.838 | 1.000 | 1.838 |
| rs9577843 | 1.830 | 1.000 | 1.830 |
| rs383353 | 1.764 | 1.000 | 1.764 |
| rs282613 | 1.717 | 1.000 | 1.717 |
| rs946006 | -1.166 | 0.000 | 0.000 |
| rs9549573 | -0.444 | 0.000 | 0.000 |
| rs12855893 | -0.294 | 0.000 | 0.000 |
| rs3024770 | -0.275 | 0.000 | 0.000 |
| rs3024771 | -0.274 | 0.000 | 0.000 |
| rs2297186 | -0.280 | 0.000 | 0.000 |
| rs3794423 | -0.316 | 0.000 | 0.000 |
| rs2270393 | -1.167 | 0.000 | 0.000 |
| rs2302758 | 3.159 | 1.000 | 3.159 |
| rs185071949 | 5.282 | 1.000 | 5.282 |
| rs117209253 | 2.859 | 1.000 | 2.859 |
| rs2274714 | 2.942 | 1.000 | 2.942 |

*
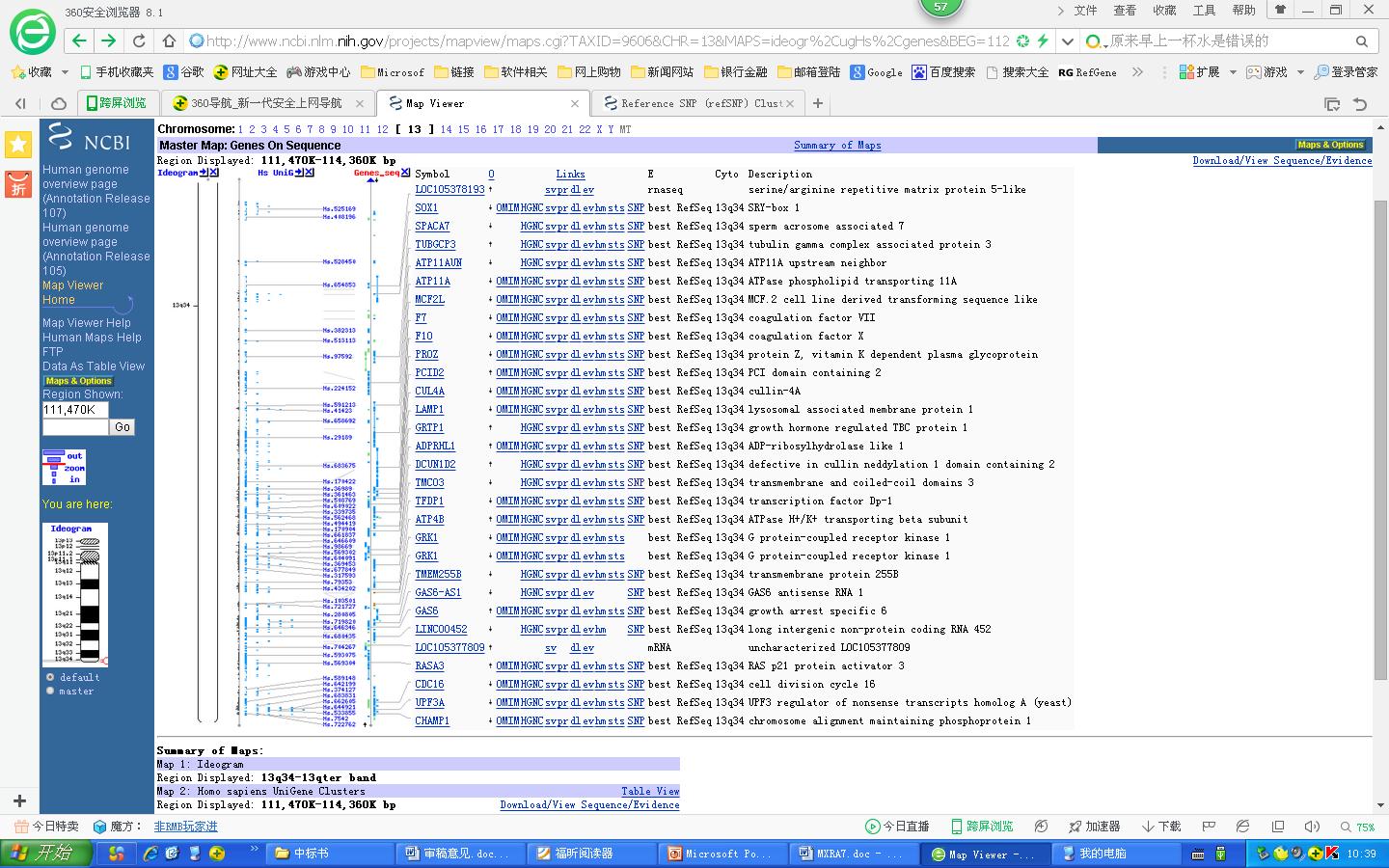
*

Supplementary Figure 1. Map of genes in the 2.9 Mb chromosome 13q34 interval.
